# Application of auto-planning in radiotherapy for breast cancer after breast-conserving surgery

**DOI:** 10.1038/s41598-020-68035-w

**Published:** 2020-07-02

**Authors:** Kunzhi Chen, Jinlong Wei, Chao Ge, Wenming Xia, Yinghua Shi, Huidong Wang, Xin Jiang

**Affiliations:** Department of Radiation Oncology, The First Hospital, Jilin University, 71 Xinmin Street, Changchun, 130021 China

**Keywords:** Radiotherapy, Biological physics

## Abstract

To evaluate the quality of planning target volume (PTV) and organs at risk (OAR) generated by the manual Pinnacle planning (manP) and Auto-Planning (AP) modules and discuss the feasibility of AP in the application of radiotherapy for patients with breast cancer. Thirty patients who underwent breast-conserving therapy were randomly selected. The Philips Pinnacle 9.10 treatment planning system was used to design the manP and AP modules for PTV and OAR distribution on the same computed tomography. A physician compared the plans in terms of dosimetric parameters and monitor units (MUs) using blind qualitative scoring. Statistical differences were evaluated using paired two-sided Wilcoxon’s signed-rank test. On comparing the plans of AP and manP modules, the conformal index (*P* < 0.01) and D_50_ (*P* = 0.04) of PTV in the AP group was lower than those in the manP group, while D_1_ was higher (*P* = 0.03). In terms of dosimetry of OAR, ipsilateral lung V_20 Gy_ (*P* < 0.01), V_10 Gy_ (*P* < 0.01), V_5 Gy_ (*P* < 0.05), and D_mean_ (*P* < 0.01) of the AP group were better than those of the manP group. Heart V_40 Gy_ and D_mean_ of all patients with breast cancer in the AP group were lower than those in the manP group (*P* < 0.01). Moreover, 12 patients with left breast cancer had the same results (*P* < 0.01). The MU value of the intensity-modulated radiation therapy module designed using two different methods was higher in the AP group than in the manP group (*P* = 0.32), although there was no statistical significance. The AP module almost had an equal quality of PTV and dose distribution as the manP module, and its OAR was less irradiated.

## Introduction

Breast cancer is one of the malignant tumors with the highest incidence in women and causes great harm to women’s health. In China, the number of patients increases annually, and the age of onset gradually decreases^1^. Breast cancer accounts for 11.6% of all cancers, ranking second according to the Global Cancer Epidemiology Statistics 2018 data^2^. In China, breast cancer is the most important tumor that endangers the health of the female population, with an age-standardized rate of 21.6 cases per 100,000 women. The first diagnosis and mortality rates of breast cancer in China are 12.2% and 9.6%, respectively, annually, ranking the sixth in the cause of cancer-related death among women in China^3,4^.

**Figure 1:**
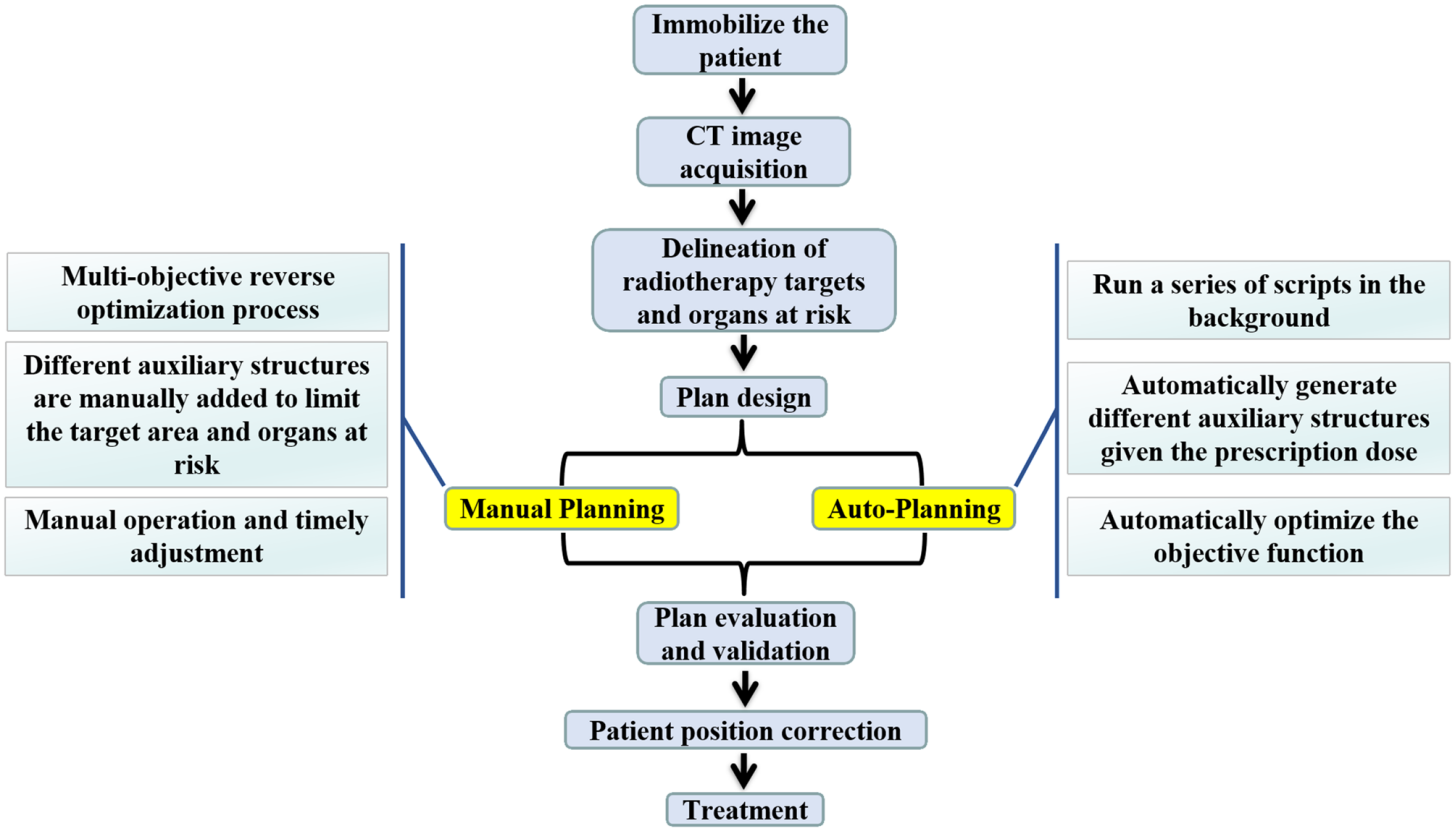
The process and characteristics of the Auto-Planning and the manual Pinnacle planning.

Presently, intensity-modulated radiation therapy (IMRT) has been widely used in radiotherapy after breast-conserving surgery^5,6^. IMRT can increase the dose in the target area, decrease the dose to organs at risk (OAR), and effectively improve the tumor control and patient survival rates^7,8^. However, IMRT planning is time-consuming and labor-intensive. Moreover, since IMRT plan optimization can be affected by multiple factors, the quality of the finalized plans varies^9,10^. Auto-Planning (AP), as a new optimization method of intensity adjustment plan, runs a series of scripts in the background to automatically generate different auxiliary structures given the prescription dose and automatically optimizes the objective function to achieve the expected results^11^. The process and characteristics of both the AP and the manP are described in Fig. 1.

In this study, 30 patients who underwent breast-conserving surgery, followed by radiotherapy for early breast cancer, were selected to plan using both manual Pinnacle planning (manP) and AP module in the Philips Pinnacle 9.10 treatment planning system (TPS). By comparing the differences in dosimetric parameters between the two plans, the feasibility of the application of AP for postoperative IMRT planning will be discussed.

## Materials and methods

### Patients

Thirty patients were randomly selected from female patients with breast cancer treated in our hospital from January 2018 to December 2018 after breast-conserving surgery. All patients received preventive radiotherapy with 50 Gy in 25 fractions prescribed to the planning target volume (PTV). Patient age distribution ranged from 33 to 67 years, with an average age of 42.3 ± 3.1 years. There were 18 cases of right breast cancer and 12 cases of left breast cancer. According to TNM clinicopathological stage (6th edition of the The American Joint Committee on Cancer), all patients were T_1–2_N_0–1_M_0_, including nine patients in T_1_N_0_M_0_ stage, 10 in T_1_N_1_M_0_ stage, 8 patients in T_2_N_0_M_0_ stage, and 3 in T_2_N_1_M_0_ stage.

### Image data and position fixation

We immobilized the patient with a special breast bracket from CIVICO. Then, the patient was simulated according to the following procedures: (1) The patient was in the supine position. (2) The head of the patient is supported by a B–F-type transparent plastic stiff pillow. (3) The affected side of the patient’s arm was placed on the stent, and the affected side’s hand holds the overhead fixation rod. (4) The patient was asked to grasp the fixed bar placed on the contralateral side of the head with the intact arm. The breast area that needs irradiation postoperatively should be fully exposed to the radiation field. We used a 24-row spiral Siemens computed tomography (CT) scan with a total scanning aperture of 800 mm in the superior–inferior direction and a slice thickness of 5 mm, and then images were uploaded to Philips Pinnacle 9.10 TPS for 3D image reconstruction.

### Delineation of radiotherapy targets and OAR

According to the National Comprehensive Cancer Network guidelines 2018 and the report no. 9804 of the Radiation Therapy Oncology Group^12^, the clinical target volume (CTV) was delineated in TPS, including intact breast tissue and tumor bed on the affected side. The specific target range of radiotherapy is as follows: (1) The inner boundary is 10 mm away from the lateral margin of the sternum. (2) The lateral boundary extends 5 mm outward to the breast gland tissue visible in the CT image. (3) The upper boundary extends 20 mm outward to the uppermost edge of the breast gland. (4) The lower boundary extends 20 mm outward to the lower edge of the breast visible in the CT image. (5) The anterior boundary extends 3 mm subcutaneously, and the posterior boundary extends to the inner edge of the chest wall and junction of the lung. Then, a three-dimensional 5-mm margin was added to the CTV to obtain the PTV. The posterior boundary expands outward to the edge of the lung tissue but does not contain the lung, and the skin retracts inward 4 mm from the subcutaneous area. Next, we delineated OARs, including the skin, ipsilateral and contralateral lung, heart, and ipsilateral breast.

### Prescription dose of PTV and dose limitation for OAR

The dosages of PTV and OAR were constrained as follows:

- PTV: 95% PTV > 50 Gy.
- Ipsilateral lung: V_20 Gy_ < 20%, V_10 Gy_ < 25%, V_5 Gy_ < 35%
- Heart: V_40 Gy_ < 30%, V_30 Gy_ < 40%, D_mean_ < 10 Gy.
- Contralateral breast: D_1_ < 5 Gy.

### Plan design

The field design of both AP and manP adopts the mixed intensification technology; that is, 70% of prescriptions are applied in the three-dimensional conformal radiotherapy (3D-CRT) technology. The shooting field direction was two angles along the internal tangent of the PTV; then, the tangential field was arranged for penetrating irradiation. After using the conformal multiple-leaf collimator, PTV expanded outward by 5 mm in each direction and 20 mm in the direction of the skin surface. The other 30% of prescriptions use direct machine parameter optimization algorithms for reverse optimization. The beam direction is the optimum line plan to outreach (5° to 10°). The subfield area of the shooting field is at least 7 cm^2^. The minimum number of hops in the subfield is 7 monitor units (MU), and the maximum number of optimization iterations is 100.

The PTV optimization objective of manP is 95% PTV > 50 Gy, and the optimization target of all OAR is the minimum value of the limited value. After the optimization result is obtained, it can be adjusted to achieve the lowest dose of the OAR. The PTV optimization target of AP is 50 Gy. OAR wait for the first result; then, it is decreased by between 1 and 3% depending on the actual dose. The weight for automatic optimization is selected as Medium.

### Evaluation index of dosimetry

According to the International Commission on Radiation Units and Measurements (ICRU) number 84 report, the dose distribution of PTV and the dose to OAR were evaluated according to the dose volume histogram (DVH), and the analysis indexes were as follows. (1) Analysis indexes of PTV include D_1_, D_50_ and D_98_, homogeneity index (HI), and conformal index (CI). HI = (D_2_–D_98_)/D_50_, in which D_2_ is the dose received by 2% target volume and the rest by analogy. CI = (V_t.ref_ /V_t_) × (V_t.ref_/V_ref_), in which V_t_ is the volume of PTV, V_t.ref_ is the volume of PTV wrapped around the isodose curve of prescription dose (50 Gy), and V_ref_ is the volume of all areas wrapped around the isodose curve of prescription dose (50 Gy). The closer the HI value is to 0 and the closer the CI value is to 1 indicate that the dose uniformity and conformability in the target area are better^13,14^. (2) The evaluation indexes of OAR include heart V_40 Gy_, V_30 Gy_, and D_mean_; maximum dose of contralateral breast D_max_; and average dose of ipsilateral lung V_5 Gy_, V_10 Gy_, V_20 Gy_, and D_mean_. (3) MU is also the main index of our evaluation. All plans are composed of three-dimensional conformal plans and intensity-modulated plans, and the conformal plans of the two groups are consistent. In this study, only the MU of the intensity-modulated plans is compared.

### Statistical analysis

The experimental data in this study were preliminarily sorted using office software and then statistically analyzed using PASW Statistics 22 and SPSS 22.0. The measurement data were expressed as mean ± standard deviation. If the comparison between the manP and AP groups conforms to the normal distribution, we will use the paired t-test, and if the comparison does not conform to the normal distribution, we will use the paired two-sided Wilcoxon’s signed-rank test. The test level was α = 0.05.

### Statement

All experimental protocols were approved by the Ethics Committee of the First Hospital of Jilin University. Informed consent requirement have been obtained from all subjects. All research was performed in accordance with relevant guidelines and regulations.

## Results

### Dose distribution in the target area of radiotherapy

Dose distributions are shown in Table 1. The planned PTV in both groups reached 95% of the volume and was irradiated at 100% of the prescribed dose, meeting the needs of clinical treatment. D_1_ in the AP group was higher than that in the manP group and was statistically significant (t = 2.25, *P* < 0.05), while D_50_ and CI in the manP group were higher than those in the AP group and were statistically significant (t = 2.25, *P* < 0.05; t = 4.50, *P* < 0.01). However, the D_98_ in the AP group was higher than in the manP group, without statistical significance. The HI was almost equal between the two plans (t = − 0.44, *P* = 0.66), without statistical significance**.**

**Table 1:** Dosimetry comparison of the PTV of AP and manP (mean ± SD) (cGy).

PTV	n	AP	manP	t	*P*-value
D_50_	30	5,143.2 ± 24.88	5,153.7 ± 25.57	2.25	**0.04**
D_98_	30	4,892.00 ± 25.55	4,889.63 ± 20.58	0.91	0.37
D_1_	30	5,299.10 ± 37.34	5,286.01 ± 17.46	2.25	**0.03**
CI	30	0.74 ± 0.06	0.76 ± 0.05	4.50	**0.00**
HI	30	0.07 ± 0.1	0.07 ± 0.01	− 0.44	0.66

### Dose to OAR

Doses to OAR are shown in Table 2. In the two groups of OAR, V_20 Gy_, V_10 Gy_, V_5 Gy_, and D_mean_ in the ipsilateral lung were lower in the AP group than in the manP group (t = 14.75, *P* < 0.01; t = 18.60, *P* < 0.01; t = 3.61, *P* < 0.05; t = 6.56, *P* < 0.01), with statistical significance. D_mean_ in the contralateral lung was also lower in the AP group than in the manP group (t = 5.88, *P* < 0.01). Among the 30 randomly selected patients, 12 had left breast cancer. Heart V_40 Gy_ and D_mean_ of all patients with breast cancer in the AP group were lower than those in the manP group (t = 2.64, *P* < 0.05; t = 4.07, *P* < 0.01), with statistical significance. Patients with left breast cancer have the same results (t = 3.22, *P* < 0.01; t = − 7.88, *P* < 0.01). However, heart V_30 Gy_ of all patients with breast cancer in the AP group was lower than that in the manP group (t = 1.6, *P* = 0.12), without statistical significance. Patients with left breast cancer also have the same results (t = 1.67, *P* = 0.12). D_max_ of the contralateral breast in the AP group was lower than that in the manP group (t = 0.86, *P* = 0.40), without statistical significance.

**Table 2:** Dosimetry comparison of OAR of AP and manP (mean ± SD).

OAR	n	AP	manP	t	*P*-value
**Ipsilateral lung**					
V_20_ (%)	30	10.83 ± 3.70	11.67 ± 3.74	14.75	** < 0.01**
V_10_ (%)	30	15.9 ± 4.25	17.61 ± 4.33	18.6	** < 0.01**
V_5_ (%)	30	22.26 ± 4.78	24.73 ± 5.10	3.61	** < 0.05**
D_mean_ (cGy)	30	603.56 ± 166.41	642.33 ± 168.08	16.56	** < 0.01**
**Contralateral lung**					
D_mean_ (cGy)	30	18.73 ± 3.44	19.10 ± 3.57	5.88	** < 0.01**
**Heart of all patients**					
V_40_ (%)	30	0.53 ± 0.88	0.58 ± 0.96	2.64	** < 0.05**
V_30_ (%)	30	0.87 ± 1.35	1.12 ± 1.95	1.6	0.12
D_mean_ (cGy)	30	149.02 ± 122.22	170.19 ± 134.87	4.07	** < 0.01**
**Heart of patients with left breast cancer**					
V_40_ (%)	12	1.34 ± 0.95	1.46 ± 1.01	3.22	** < 0.01**
V_30_ (%)	12	2.18 ± 1.30	2.81 ± 2.20	1.67	0.12
D_mean_ (cGy)	12	282.50 ± 77.07	318.63 ± 80.87	− 7.88	** < 0.01**
**Contralateral breast**					
D_max_ (cGy)	30	294.45 ± 232.48	320.46 ± 246.24	0.86	0.4

**Figure 2:**
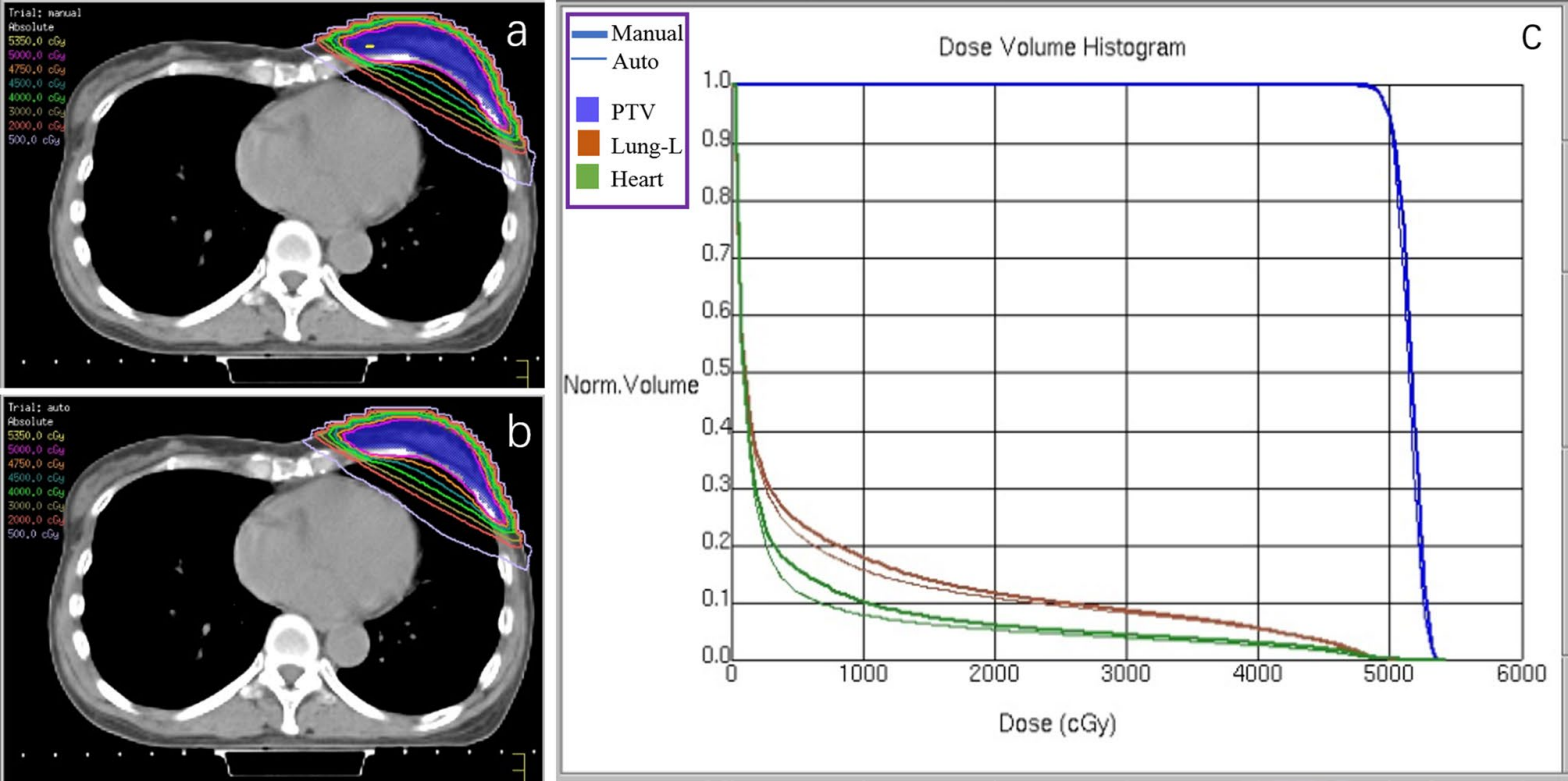
Dose distribution for manP (**a**) and AP (**b**) and comparison between the two DVHs (**c**).

In Fig. 2, the dose distribution in the manP and AP groups with the comparison between the two DVHs is reported for a representative patient.

### MU of the plan

The MU of the intensity-modulated plan designed by the two different methods was higher in the AP group than in the manP group (t = − 1.01, *P* = 0.32), but there was no statistical significance. Data are summarized in Table 3.

**Table 3:** Dosimetry comparison of the MU of AP and manP (mean ± SD).

Group	n	MU
AP	30	104.37 ± 15.70
manP	30	100.97 ± 10.11
t		− 0.14
*P*		0.32

## Discussion

Breast cancer is a malignant tumor developing in the epithelial tissue of the breast, ranking first in the list of malignant tumors in women^15^. Radiotherapy after breast-conserving surgery can significantly reduce the local recurrence rate and improve the effective survival rate of patients. With the improvement in people’s aesthetic consciousness, the requirements of breast appearance after radiotherapy for breast cancer have also improved^16–18^. The application of IMRT combined with 3D-CRT can provide the postoperative breast tissue with adequate dose of preventive irradiation, while effectively limiting the OARs irradiation. Therefore, IMRT can help reduce the development of corresponding complications and reduce the risk of secondary cancer caused by radiation. Moreover, we can obtain better dose conformal degree and uniformity in the target area of radiotherapy, which can greatly reduce the local fibrosis of the breast tissue and gland atrophy, so as to meet patients’ pursuit of beauty^19^.

Presently, the design of an intensity-modulated plan is based on different target areas of radiotherapy and positions of OAR, manually adding some dosimetric auxiliary structures, then setting different dosimetric parameters, and finally using the planning system to reverse optimize the algorithm. Based on the results, we repeatedly modified parameters and set different dosimetric auxiliary structures. This is a process of repeated modifications to obtain a treatment plan that meets clinical requirements. The manP design process is tedious and time-consuming and will introduce human errors. Besides, manP is often not repetitive. For comparison, AP with the Pinnacle 9.10 TPS was designed with fewer affected factors and no additional dosimetric ancillary structure required. According to the dose distribution of PTV and OAR in real time in the optimization process, it will automatically generate a series of dosimetric auxiliary structures and perform repeated optimization calculations on the target function to achieve the expected goal^20^.

According to the ICRU number 84 report, the evaluation tool for PTV is the DVH. Its indicators mainly include HI and CI. The closer the CI is to 1, the better the dose curve wraps PTV, while the closer the HI is to 0, the better the dose uniformity. It can be noted from the results that the CI and D_1_ in the manP group are better than that in the AP group. This is because manP not only makes a RING on PTV to improve the conformability of target dose but also deals with high-dose hot spots and low-dose cold spots. However, there was no relevant option to limit the dose conformal degree and uniformity of PTV in the parameters of AP design, which resulted in higher D_1_ and D_98_ than those in the manP group. Although there is a slight difference between the two plans, their dose distribution in PTV alone can meet the needs of clinical treatment.

Both V_20 Gy_ and D_mean_ in the lung tissue are independent factors affecting the development of interstitial pneumonia^21^, which significantly affects the long-term survival and quality of life of patients^22^. Additionally, the D_mean_ of the heart cannot be ignored in the long-term survival of patients with cardiac function^23^. In terms of the dose received by the OAR in the two groups, the V_20 Gy_, V_10 Gy_, V_5 Gy_, and D_mean_ of the ipsilateral lung and V_40 Gy_ and D_mean_ of the heart in the AP group were all superior to those in the manP group. In our study, we also performed a separate analysis of the dose received by the heart in patients with left breast cancer and obtained the same results as in all patients. In the evaluation index of dosimetry, MU is also an important index we need observe. The MU of the intensity-modulated plan designed by the two different methods was higher in the AP group than in the manP group (t = − 1.01, *P* = 0.32), but there was no statistical significance. When selecting AP parameter weight, the parameter weight of each OAR had only four options included: Low, Medium, High, and Constrain. In this study, we only selected Medium. Therefore, if other weights are selected or different weights are combined, OAR will be exposed to less dose radiation. This is something that this study lacks, and it is also worthy of our deep consideration and further discussion.

Currently, there are several ongoing studies on AP. Wei et al. reported that the OAR of the AP group after right modified radical mastectomy was also significantly better than that of the manP group^24^. Marrazzo et al. pointed out that AP can significantly improve the dose coverage of PTV and reduce the dose to OAR when using the volume intensity-modulated technique designed by AP^25^. Purdie et al. shown that when using an automated technique for two-field tangential breast IMRT treatment planning, the AP was dosimetrically equivalent to the clinical plans when scored for target coverage and lung and heart doses^26^. Studies on other cancers have also shown that AP can effectively improve the dose coverage of PTV and reduce the dose of OAR^11,27–30^. However, our study is only consistent with the findings of other scholars on OAR, but the situation of PTV has not been improved. This is largely dependent on the plan of our study, which is the mixed irradiation technology of 3D-CRT and IMRT, among which the prescription of 3D-CRT plan accounts for 70% of the total prescription. Therefore, the HI and CI adjustable space of PTV is only 30%. Finally, the AP group did not improve the HI and CI of PTV compared with the manP group.

The evaluation of the radiotherapy plan is as comprehensive as the treatment of breast cancer. It is necessary to consider not only the dose coverage and evenness index of PTV but also the dose of OAR around PTV. When focusing on PTV, the dose curve distribution is ideal, and the cure rate of the tumor will be greatly improved. However, concurrently, it will also increase the development of various complications and reduce the quality of life of patients. However, when focusing on OAR, in the case of better organ protection, the therapeutic effect on the tumor may be slightly affected^31^. Although the PTV coverage of the manP group was superior to that of the AP group, the differences between them were small. In contrast, the AP group’s plan outperformed that of the manP group in terms of OAR dose although both can meet the needs of clinical treatment.

This study also has some limitations. It does not fully consider the influence of OAR weight on dose distribution of PTV and OAR.

## Conclusions

AP as a design approach based on script automation plans, when compared with manP, has the following advantages: (1) it does not need several auxiliary structures of organs dose and crisis. (2) It does not require large amounts of standardized database support and millions of financial support like artificial intelligence software. (3) The plan designed by AP can be fully used in radiotherapy after breast-conserving surgery, which can reduce the work burden of the plan designer to a certain extent, and the quality of the treatment plan can also be guaranteed to a certain extent. On the contrary, for primary hospitals, it can solve heavy work burden caused by insufficient medical physicist, which is a relatively effective means and approach with low economic cost at present.
